# Subacute and chronic proteomic and phosphoproteomic analyses of a mouse model of traumatic brain injury at two timepoints and comparison with chronic traumatic encephalopathy in human samples

**DOI:** 10.1186/s13041-022-00945-4

**Published:** 2022-07-18

**Authors:** Alexander Morin, Roderick Davis, Teresa Darcey, Michael Mullan, Benoit Mouzon, Fiona Crawford

**Affiliations:** 1 Roskamp Institute, Sarasota, USA; 2 The Open University, Milton Keynes, UK; 3 The James A Haley Veterans’ Administration, Tampa, USA

## Abstract

Repetitive mild traumatic brain injury (r-mTBI) is the most widespread type of brain trauma worldwide. The cumulative injury effect triggers long-lasting pathological and molecular changes that may increase risk of chronic neurodegenerative diseases. R-mTBI is also characterized by changes in the brain proteome, where the majority of molecules altered early post-TBI are different from those altered at more chronic phases. This differentiation may contribute to the heterogeneity of available data on potential therapeutic targets and may present an obstacle in developing effective treatments. Here, we aimed to characterize a proteome profile of r-mTBI in a mouse model at two time points – 3 and 24 weeks post last TBI, as this may be a more relevant therapeutic window for individuals suffering negative consequences of r-mTBI. We identified a great number of proteins and phosphoproteins that remain continuously dysregulated from 3 to 24 weeks. These proteins may serve as effective therapeutic targets for sub-acute and chronic stages of post r-mTBI. We also compared canonical pathway activation associated with either total proteins or phosphoproteins and revealed that they both are upregulated at 24 weeks. However, at 3 weeks post-TBI, only pathways associated with total proteins are upregulated, while pathways driven by phosphoproteins are downregulated. Finally, to assess the translatability of our data, we compared proteomic changes in our mouse model with those reported in autopsied human samples of Chronic Traumatic Encephalopathy (CTE) patients compared to controls. We observed 39 common proteins that were upregulated in both species and 24 common pathways associated with these proteins. These findings support the translational relevance of our mouse model of r-mTBI for successful identification and translation of therapeutic targets.

**Supplementary Information:**

The online version contains supplementary material available at 10.1186/s13041-022-00945-4.

## Introduction

Repetitive mild traumatic brain injury (mTBI) is a recognized risk for long term neurodegenerative disabilities such as Alzheimer’s Disease (AD), Chronic Traumatic Encephalopathy (CTE), and other forms of dementia [1–3]. Numerous studies of repetitive mild TBI (r-mTBI), especially in contact sport players, have revealed a months-to-years-long period between the injury and the onset of cognitive deficits [4–6]. During this time, the brain undergoes a complex array of neuropathological changes varying from axonal injury, vascular damage and inflammation to neurodegeneration [4, 7–9]. Some of these processes are specific to only acute or chronic time points while others are present in both. Understanding such temporal heterogeneity may be crucial for therapeutic target identification.

Mass-spectrometry (MS) based proteomics and phosphoproteomics allow for the unbiased investigation of large numbers of proteins and associated biological processes and comparison of datasets from TBI versus control samples could identify potential therapeutic targets. However, to date, the number of published studies on mTBI proteomics, and especially phosphoproteomics, remains scarce. In their 2018 review, Sowers et al. outlined only ten TBI-related published proteomic studies, five of which were conducted in human samples (TBI, AD or CTE), two in in-vitro models and three in in-vivo animal models [10]. Among them, only one study has focused on mTBI in animal models [11] and none in human samples. Between 2010 and 2017, an independent meta-analysis by Ganau et al. identified only 16 omic papers on TBI, 8 clinical and 8 animal studies, ranging by the type of analysis, injury severity, animal species, and other variables [12]. Phosphoproteomics represents an even less well investigated area with only a single study conducted in an mTBI mouse model [13]. Combined, the published data do not present a consistent message, owing to the diverse models of TBI under investigation, and differences in timing of assessment post-injury, species, animal genotypes, and other factors. Altogether, the heterogeneity of these variables plus the existing complexity of mTBI pathology preclude clear comparison between different omic datasets.

Here, we designed a study that addresses the temporal changes of r-mTBI using proteomic- and phosphoproteomic-based approaches within a single animal model. Mice (hTau) received 5 closed-head injuries over 9 days, and cortical tissues were collected at either 3 or 24 weeks post last injury, representing subacute and chronic periods, respectively [14, 15]. In our previous studies, this r-mTBI model results in sustained lifelong behavioral deficits, glial activation, and tau phosphorylation up to 24 months post last injury [16, 17]. The choice of hTau mice, which express the six isoforms of human tau, is to better mimic tauopathies involved in chronic forms of neurodegeneration post TBI, as we have previously not observed persistence of any TBI-dependent changes in Tau in wild type mice [18]. To validate the clinical translatability of our model, we compared the murine proteome with previously acquired human CTE proteomic data. There is a lack of open-access raw datasets from human TBI, but a publicly available dataset from cortices of human CTE cases is available [19], and this raw dataset of protein changes in human CTE samples (ProteomeXchange) allowed us to analyze it in the same way as we did with our own data. We hypothesize that cellular mechanisms found to be similarly altered in human samples and in our mouse model may represent translational therapeutic targets which can be further validated and pursued in our model.

## Results

### Commonly altered proteins and phosphoproteins post-TBI

**Fig. 1:**
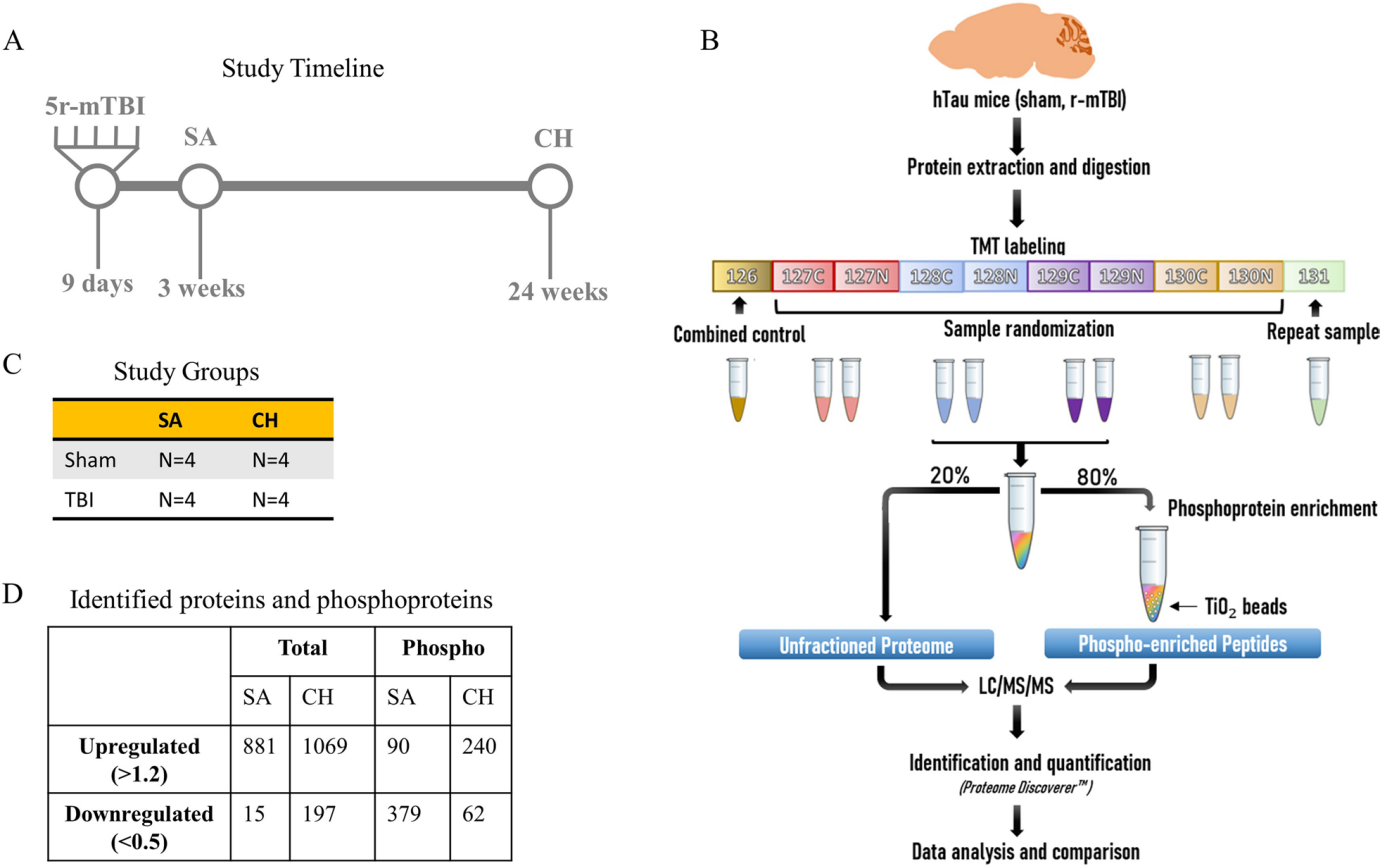
**A** Timeline of the study. Mice received 5 consecutive mTBI over 9 days (48 h interval between the injuries) at 3 months of age. They were further divided into 2 cohorts: subacute (SA) and chronic (CH). SA mice were euthanized at 3 weeks after the last injury, CH mice at 24 weeks. **B** Proteomic/phosphoproteomic design. Mouse cortices of both cohorts were processed using TMT labeling. Phospho-enrichment was performed using TiO_2_ beads. **C** The number of mice (n = 4) in each group. **D** Total number of identified proteins. Detected proteins and phosphoproteins were described as up- or down-regulated using thresholds for abundance ratio (a.r.) > 1.2 or < 0.5, respectively

Mouse cortices were extracted at 3 weeks (sub-acute—SA) or 24 weeks (chronic—CH) after the last injury or sham procedure (Fig. 1A). Each group had n = 4 mice (Fig. 1C). Proteomic and phosphoproteomic analyses were conducted as shown in Fig. 1B. Overall, 4511 total proteins and 861 phosphoproteins were identified in both cohorts. If a protein was not detected in at least one TBI sample or one Sham sample, it was excluded from further analysis. Those with abundance ratios (a.r.) > 1.2 or < 0.5 were selected for further analysis to represent upregulation or downregulation, respectively [20, 21]. The SA cohort was characterized by 881 up- and 15 down-regulated proteins and 90 up- and 379 down-regulated phosphoproteins, while the CH mice had 1069 up- and 197 down-regulated proteins and 240 up- and 62 down-regulated phosphoproteins (Fig. 1D). Raw data is provided as a supplement (Additional file 1).

**Fig. 2:**
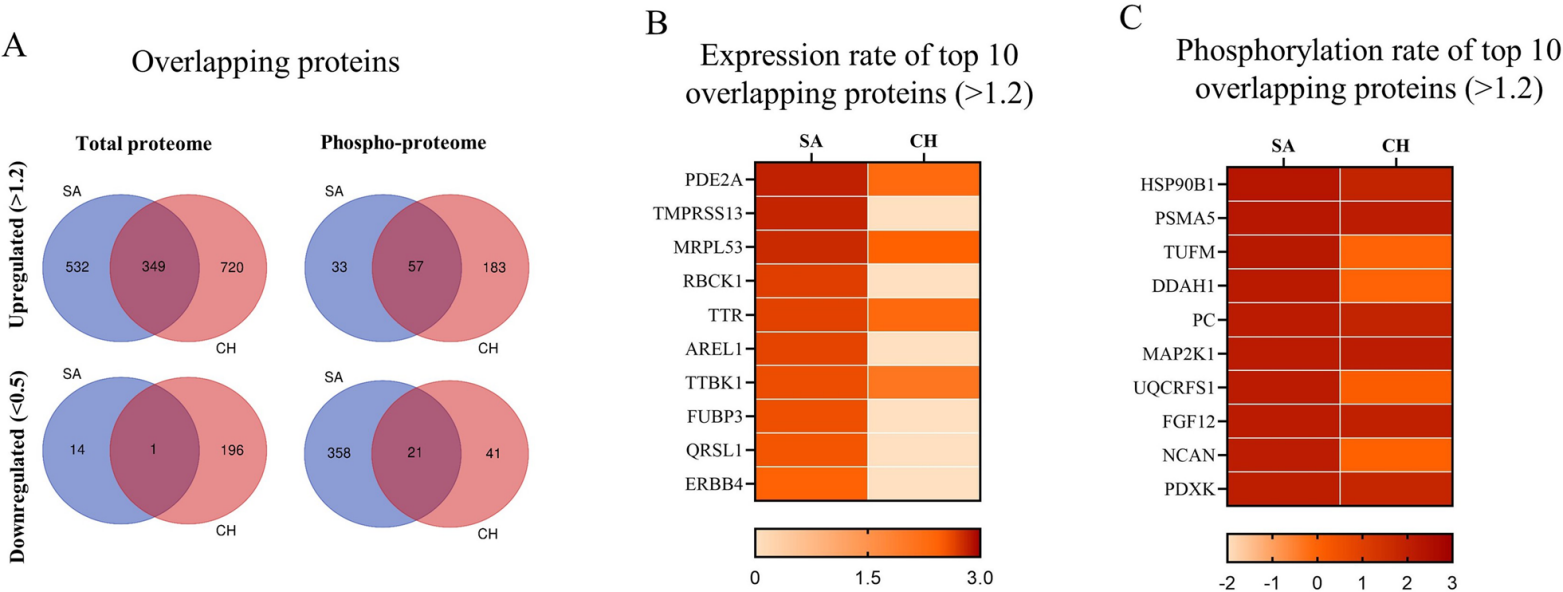
**A** Number of total proteins and phosphoproteins identified in SA and CH cohorts stratified by their a.r. as up- (> 1.2) or downregulated (< 0.5). Venn diagrams show the overlapping proteins between two cohorts. **B** Heatmap of the top 10 commonly upregulated total proteins stratified by the log10 values of a.r. **C** Heatmap of the top 10 commonly upregulated phosphoproteins stratified by the log10 values of a.r

To demonstrate persistent changes from 3 to 24 weeks post-TBI, we identified proteins which were similarly altered in response to r-mTBI in both cohorts of mice. Overall, both SA and CH cohorts showed an upregulation of 349 total and 57 phosphoproteins and a downregulation of 1 total and 21 phosphoproteins (Fig. 2). Given the much higher number of significantly altered proteins in the upregulated group, our analysis focused primarily on the datasets of 349 total and 57 phosphoproteins. Stratification of these proteins by their a.r. identified PDE2A (phosphodiesterase 2A) as the total protein most significantly altered in response to r-mTBI (a.r.: SA-2.83, CH-2.29), followed by MRPL53 (mitochondrial ribosomal protein L53, a.r.: SA-2.78, CH-2.45), TTR (transthyretin, a.r.: SA-2.65, CH-2.31), and TTBK1 (tau tubulin kinase 1, a.r.: SA-2.59, CH-2.06) (Fig. 2B). Phosphorylated proteins with the greatest change in expression in TBI vs sham which were common to both cohorts included HSP90B1 (heat shock protein 90 beta family member 1), PSMA5 (proteasome subunit alpha type-5), PC (pyruvate carboxylase), MAP2K1 (mitogen-activated protein kinase 1), FGF12 (fibroblast growth factor 12), and PDXK (pyridoxal kinase) (Fig. 2C).

**Fig. 3:**
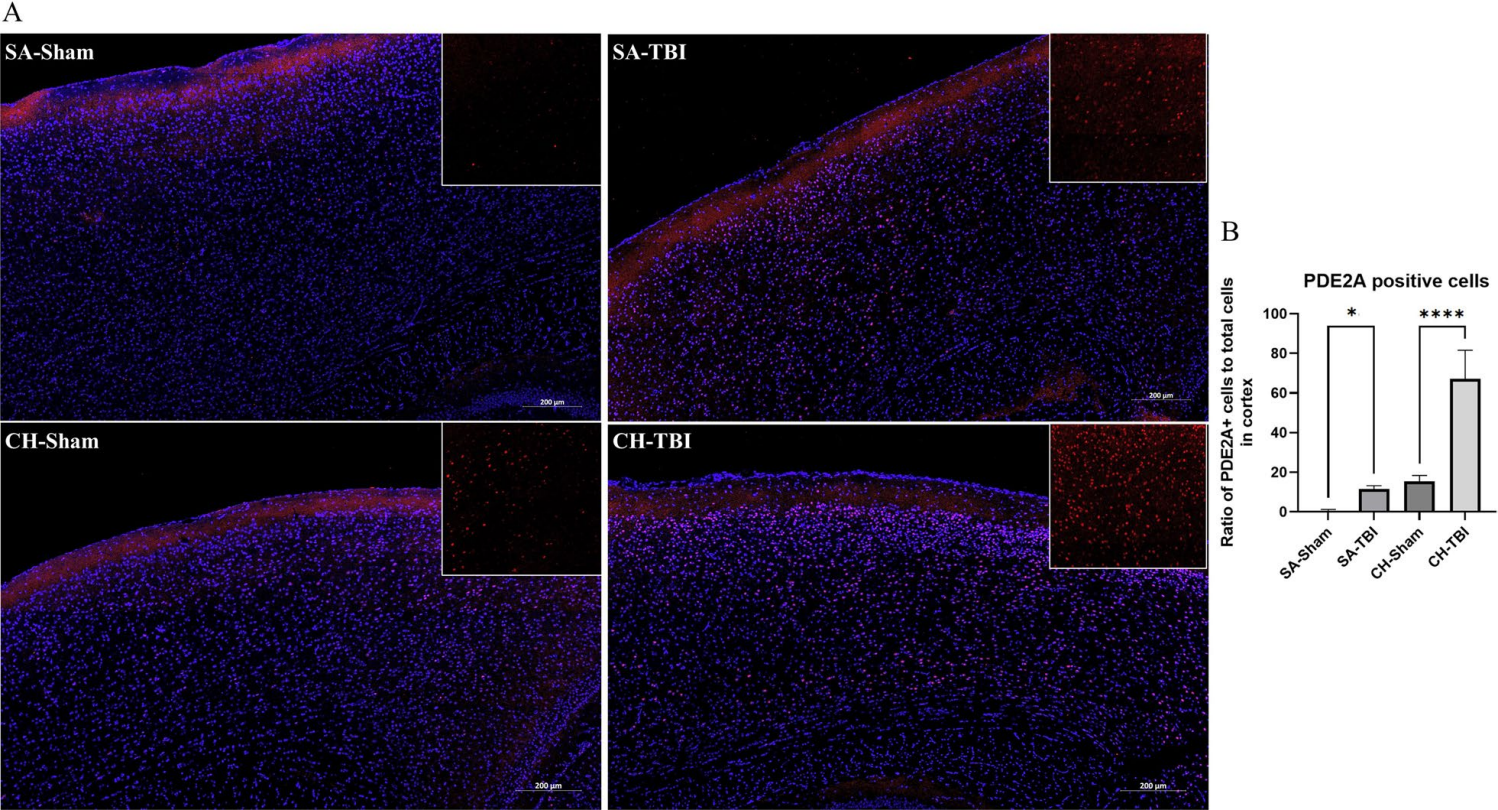
**A** Immunofluorescent staining for PDE2A in the cortex. **B** The ratio of the PDE2A positive cells to the total number of cells (DAPI) shows an increase in TBI vs sham in both SA (p < 0.05) and CH cohorts (p < 0.001). Data were analyzed using two-way ANOVA

As a proof of concept, we selected a protein showing a significant level of change in both TBI cohorts compared to shams, PDE2A, for the antibody-based validation. Quantitative analysis of immunofluorescent staining of PDE2A (Fig. 3A) was performed across somatomotor, anterior cingulate, and retrosplenial areas corresponding to the parts of cortex that were used for the proteomic analyses. It demonstrated an increase in PDE2A-positive cells in both SA (p < 0.05) and CH (p < 0.001) r-mTBI cohorts compared to their respective shams (Fig. 3B).

### Commonly altered canonical pathways post-TBI

**Fig. 4:**
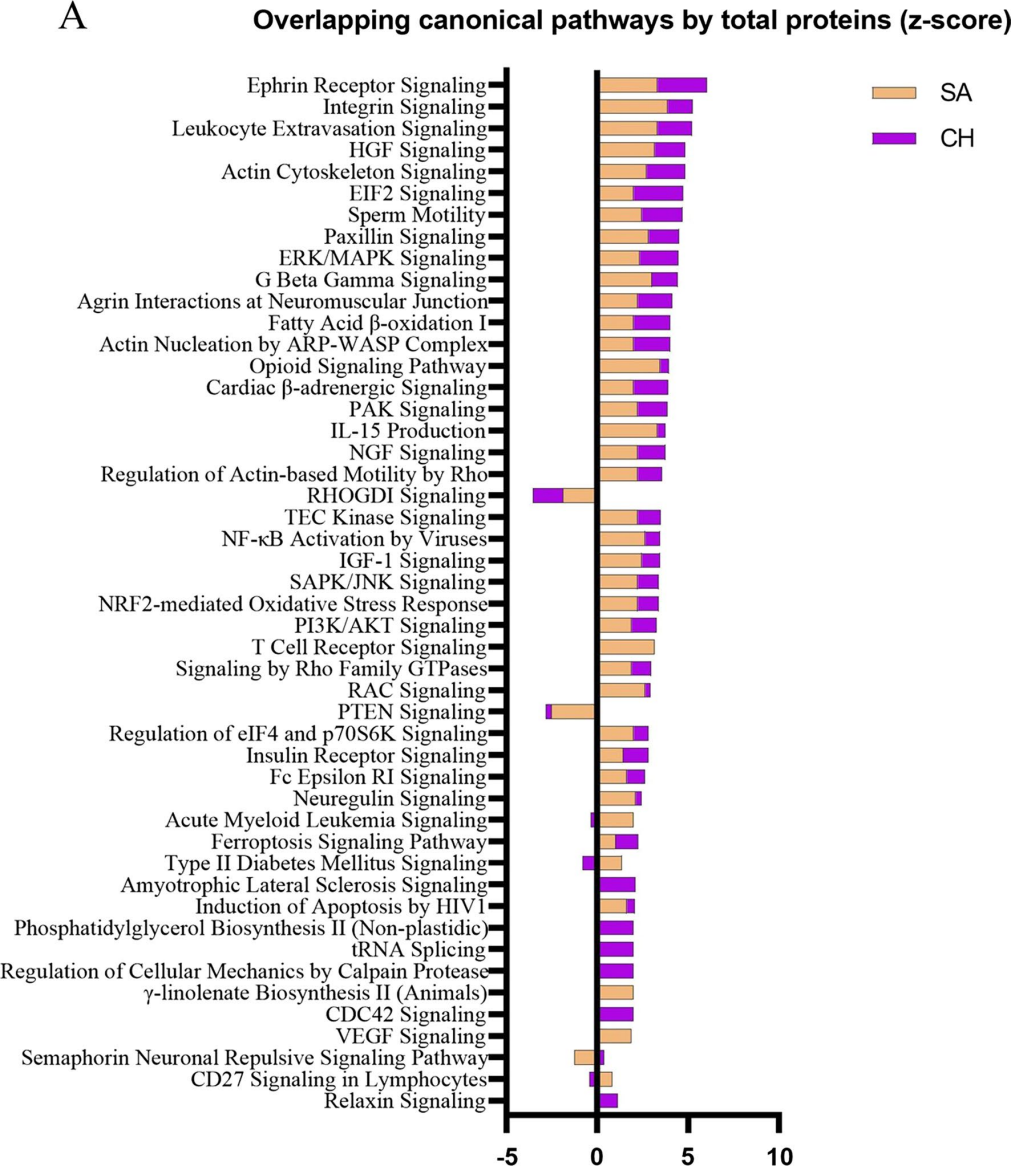

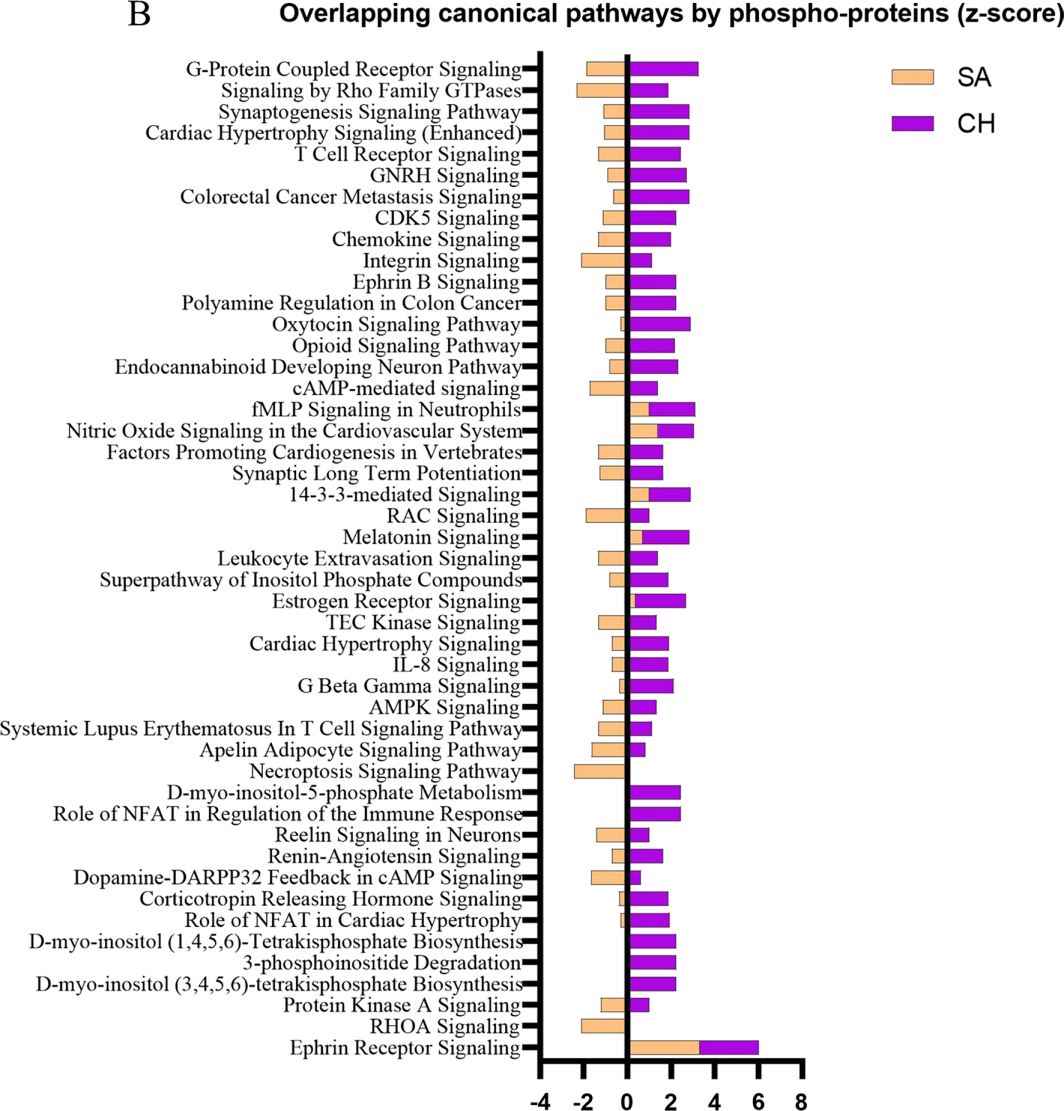
Bar graphs representing activation state (z-score) of canonical pathways by total (**A**) or phospho-proteome (**B**). Upregulation is described with a positive z-score, downregulation with a negative z-score

In addition to the comparison of individual proteins, we further decided to compare canonical pathways between the cohorts due to the possibility that the same pathways could be activated through TBI-dependent modulation of different proteins. The activation state was calculated using z-score where a positive value infers a pathway’s upregulation, and a negative value infers downregulation. A comparison analysis of responses in canonical pathways resulting from expression changes in total proteins revealed a pattern of activation of many of the same pathways in both SA and CH cohorts (Fig. 4A). The top processes included *Ephrin Receptor Signaling (z-score: SA-3.32, CH-2.71), Integrin Signaling (z-score: SA-3.87, CH-1.39), Leukocyte Extravasation Signaling (z-score: SA-3.32, CH-1.89), EIF2 Signaling (z-score: SA-2.00, CH-2.71)* and others. From the whole list, only two pathways were identified as being inhibited in both two cohorts—*RHOGDI Signaling (z-score: SA-−* *1.89, CH-−* *1.67) and PTEN Signaling (z-score: SA-−* *2.53, CH-−* *0.30)*. In contrast, the z-scores for canonical pathways based on the phosphoprotein analyses showed almost exclusively opposite direction activation in SA versus CH cohorts, suggesting downregulation of those pathways at the SA timepoint and upregulation at the CH timepoint (Fig. 4B). A complete list of molecules constituting these pathways and of the molecules that were significantly regulated in both the SA and CH cohorts is provided in the Additional file 2 and Additional file 3: Fig. S1).

### Overlap of mouse proteome with human CTE

**Fig. 5:**
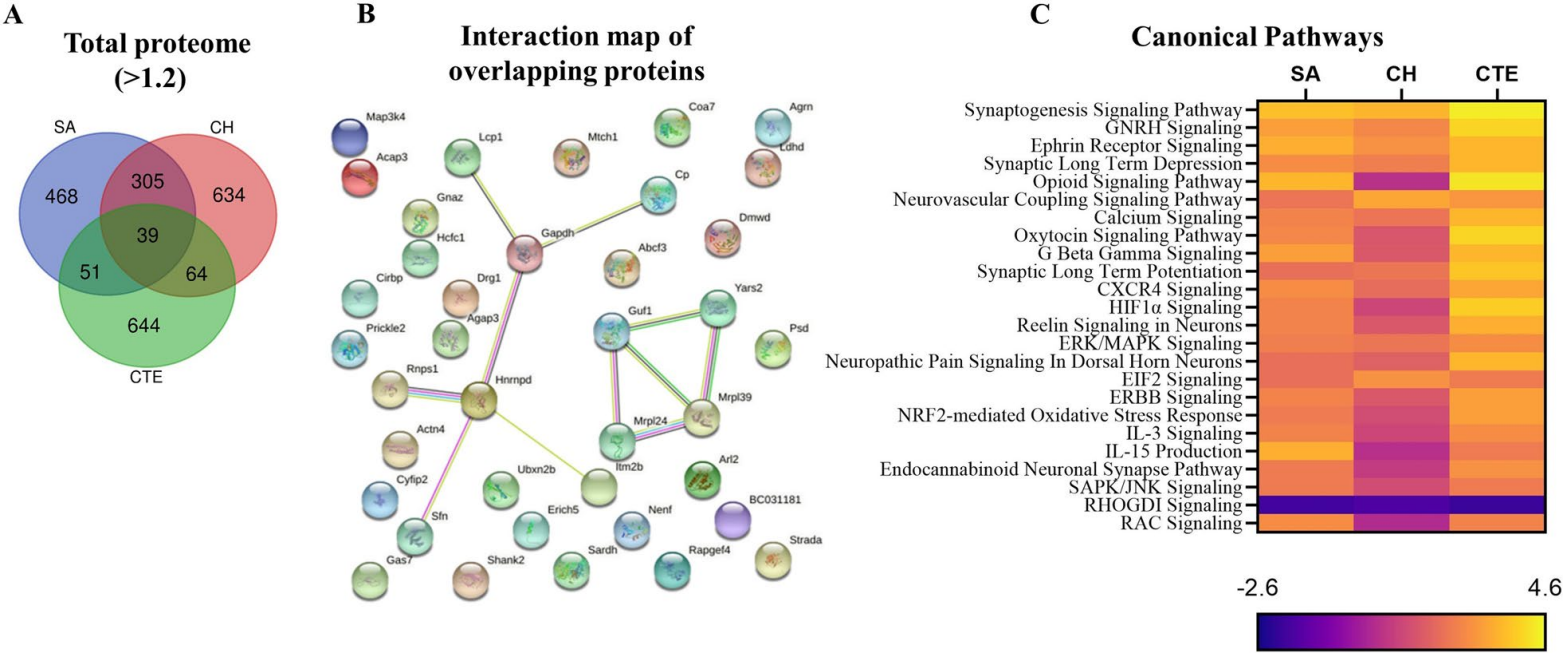
**A** An overlap of total upregulated proteins between the SA and CH cohorts in a mouse model of r-mTBI and the human CTE samples. **B** Interaction map of the 39 identified overlapping proteins (STRING 11.5, confidence score > 0.7). **C** Overlapping canonical pathways stratified by the activation z-score

In order to evaluate the relevance of our r-mTBI mouse model to human clinical chronic outcomes, we accessed a proteomic database of CTE cases (n = 11) with a history of concussions, which was available from the ProteomeXchange dataset PXD007694 [19]. The total proteome of both SA and CH cohorts was compared to that of the CTE dataset, and the overlapping upregulated proteins were identified (no overlap of downregulated proteins across all three groups was observed). During the conversion of mouse protein accession numbers to their respective proteins in the human proteome, some molecules did not have a match and were thus excluded from the comparison. Hence, the number of upregulated proteins utilized for the TBI-CTE comparison is different from the originally identified proteins as shown in Fig. 1. From the proteins that were successfully converted from mouse to human, we identified 39 proteins that were significantly upregulated in all three cohorts compared to their respective controls (Fig. 5A, B). A comparison analysis of canonical pathways predicted to be responding in the mouse models of r-mTBI and human CTE (Fig. 5C) revealed 24 common pathways. Of these, 23 pathways were upregulated in all cohorts and the remaining one was downregulated across all cohorts. The upregulated pathways included *Synaptogenesis Signaling Pathway, GNRH signaling, Ephrin Signaling, Synaptic Long-Term Depression, Neurovascular Coupling* and others*.* The single pathway downregulated in all cohorts was *RHOGDI Signaling* (z-score: SA-− 1.89, CH-− 1.67, CTE-− 2.00).

## Discussion

The goal of this study was to describe proteomic and phosphoproteomic changes common to sub-acute (SA) and chronic (CH) time points after r-mTBI that might represent the best potential targets in a heterogeneous TBI population. Overall, we showed that both SA and CH profiles share a large number of upregulated proteins, while the phosphoproteome profiles were, in general, more specific for each time-point. The SA phosphoproteome was dominated by downregulated proteins while at the CH timepoint the response to r-mTBI was largely upregulation of phosphoproteins. Only a few of the downregulated phosphoproteins in SA mice remained below the sham expression levels by the chronic time point, suggesting their specificity to sub-acute post-TBI response. Such dynamics reflect the transient nature of protein phosphorylation after r-mTBI underscoring the importance of post-TBI timing of certain pathological responses and the limitations they might place on therapeutic windows for potential interventions. However, the goal of this study was to evaluate proteins that kept consistent levels of regulation from 3 to 24 weeks after TBI and hence our analyses was primarily focused on the total proteome.

Studying proteins that are similarly regulated at different timepoints post-injury is an important undertaking. Owing to the heterogeneity of human TBI itself, as well as the potential timepoints post TBI at which intervention may be initiated, a reasonable first approach to determining therapeutic targets that could work in multiple scenarios is to identify TBI dependent changes that persist over a period of weeks or months in a preclinical model, as this could translate to clinically relevant timeframes for therapeutic intervention in the human patient population. Such an approach has been proposed as the next logical move in TBI research by several scientists [22, 23]. In their review, Kenzie et al. performed a causal-loop analysis of post-mTBI mechanisms and demonstrated a nonlinear pattern of mTBI recovery [22]. The acute phase is often characterized by cell loss and axonal damage, while at subacute and chronic phases, neuroinflammation and neuroplasticity mechanisms are prevalent [23]. Such heterogeneity is traced down to individual proteins which comprise diverse proteomic profiles at different timepoints post TBI. For example, in their study, Lizhnyak and Ottens demonstrated that at 14 days after moderate TBI, 52% of dysregulated proteins were novel compared to the proteins identified at the 2-day timepoint [23]. In our study, 72% of proteins dysregulated at 24 weeks after r-mTBI, (both up- and down-regulated) had not been identified as dysregulated at the 3-week timepoint. For effective treatment approaches it will be critical to understand the lifespan of any proposed therapeutic target. To date, many treatments have shown efficacy in preclinical models but have not been successful with clinical translation, and a contributor to this may have been insufficient interrogation of the viable therapeutic window at the preclinical stage.

So far, only a few proteomic studies have proposed potential targets in relation to timing post injury. Ojo and colleagues measured altered proteins in the cortex and hippocampus of C57BL/6J mice at 3, 6, 9 and 12 months after r-mTBI, using the same 5-hit model we utilized in our current study [24]. Among the most dysregulated processes common for all time-points were PI3K/AKT, PKA, and PPARα/RXRα signaling in the hippocampus, and PKA, GNRH, and B cell receptor signaling in the cortex. To our knowledge, the only other proteomic study that compares acute/sub-acute and chronic time points after mild TBI was conducted by Song et al. [11]. They measured temporal changes in the total proteome of Sprague–Dawley rats at 1 day, 7 days or 6 months after either a single mTBI or a 3-hit r-mTBI. In addition to demonstrating larger TBI-dependent fold-changes in protein expression, they described two upregulated proteins, PDE10A and GNAL, which did not return to the sham levels even at 6 months post-r-mTBI. PDE10A belongs to the class of phosphodiesterases, as does PDE2A, highlighted in our current study, which is a group of enzymes that hydrolyze cAMP and cGMP, terminating their signaling [25]. In the brain, they contribute to learning and memory, while in the periphery they play an important role in macrophage differentiation [26]. Inhibition of PDE2 prevents degradation of cGMP leading to enhanced long-term potentiation and improved memory [27, 28]. Hence, prolonged increased levels of PDE2 might be associated with chronic memory deficits. The same mice as used in the current paper underwent behavioral assessment at sub-acute and chronic time points [29, 30] and demonstrated r-mTBI-dependent impaired learning and memory. Using immunofluorescent imaging, we have confirmed an increased number of PDE2A-expressing cells in the cortex after r-mTBI at both 3- and 24-weeks post injury. Further validation of PDE2A as a target, and the effects of its inhibition in injured mice, will be a direction for future studies.

In addition to PDE2A, another protein that was increased at 3 and 24 weeks post injury was tau tubulin kinase 1 (TTBK1), which is known to be linked to the development of several neurodegenerative diseases such as ALS, FTLD, and AD [31]. In AD, TTBK1 phosphorylates tau at Ser422 contributing to the formation of neurofibrillary tangles [32]. Animal models support these observations showing that mice expressing human TTBK1 develop tau phosphorylation, gliosis, and spatial memory impairment [33]. In our previous study, we demonstrated hyperphosphorylation of tau at Thr231 in aged hTau at 3 weeks after the same r-mTBI model, but not in young mice [16, 29]. While no clear correlation can be made between TTBK1 and tau in our SA and CH cohorts, these independent findings highlight a possible role of tau in the chronicity of TBI pathology. One of our future goals is to investigate additional phospho-epitopes of tau that can be linked to TTBK1 activity.

An intriguing trend was observed when we created an activation profile of canonical pathways based on either phospho- or total proteins. While canonical pathways derived from the TBI-dependent changes in the total proteome were consistently upregulated between SA and CH cohorts, phospho-based pathways were largely downregulated in the SA cohort but upregulated in the CH cohort. Due to the lack of similar studies in the literature, it is hard to compare these findings with other models. When we compared our total protein data with CTE data, we found that both human and mouse data demonstrated a great number of upregulated pathways with only one downregulated process (*RHOGDI*). The large number of pathways shown to be activated in our mouse models which are also activated in CTE speaks to the relevance of our model for investigations of human TBI pathogenesis.

Our study has several limitations that are worth acknowledging. First, the use of homogenized tissue from the whole cortex may have masked detection of molecular responses that are cell specific. Future studies utilizing astroglial and microglial fractions instead of the whole tissue may reveal more specialized mechanisms involved in TBI pathogenesis, especially those related to neuroinflammation. Secondly, common caveats of proteomic studies are the biological and technical limitations. They may result in a decreased sensitivity for low abundance proteins, inaccuracies in ratio calculations, and false negative/positive results. To minimize the latter, we applied strict cutoff thresholds for identification of significant changes in protein expression, and high-stringency analysis of protein–protein interactions for canonical pathways identification. Lastly, a sample size of four mice per group presents statistical limitations, and a higher-powered study would increase the accuracy of the observed signal. However, we relied on our previous data where a similar sample size of four was used in a r-mTBI model and showed statistically significant changes in TBI-induced pathology and behavior [14, 15, 29, 30, 34]. Moreover, tissue described in the current manuscript was used in several other analyses where it demonstrated increased neuroinflammation, tauopathy, and spatial memory deficits [29, 30].

Overall, our data signify the importance of investigating not only the total proteome but also the phosphoproteome and comparing animal data with human for clinical relevance. This approach may help to identify therapeutic targets for TBI but also provides a broad timeline of the progression of TBI pathobiology. Future studies will focus on validating candidate proteins and pathways that exhibit both therapeutic and translational potential.

## Methods

### Animals

Male and female hTau mice (n = 16) 12–14 weeks old (weight 19-25 g) were sourced from Jackson Laboratories (Bar Harbor, ME). The animals were housed under standard laboratory conditions (14-h light/10-h dark cycle, 23 ± 1 °C, 50 ± 5% humidity) with free access to food and water. All procedures were carried out under Institutional Animal Care and Use Committee approval (Roskamp Institute IACUC) and in accordance with the National Institutes of Health Guide for the Care and Use of Laboratory Animals.

### Repetitive mild TBI

For sham/mTBI procedures, all animals underwent anesthesia with 1.5 mL/min of oxygen and 3% isoflurane for 3 min on a heated platform to prevent hypothermia. Mice assigned to TBI procedures were also maintained on a heating pad during the injury procedure to prevent hypothermia. The head of each animal was fixed in a stereotaxic frame, and a 5 mm blunt metal impactor was positioned midway on the sagittal suture. The injury was triggered at 5 m/s velocity and 1.0 mm depth, with a dwell time of 200 ms, using a myNeuroLab controller device (Impact One™ Stereotaxic Impactor, Richmond, IL). All TBI mice experienced short-term apnea (< 20 s) and showed no skull fractures. All animals (sham and TBI) recovered from anesthesia on a heating pad and were then returned to their cages with water and soft food access. In total, mice received 5 hits (or in the case of sham mice, 5 anesthesias of the same duration as TBI mice), one every 48 h for the duration of 9 days as shown in Fig. 1A. After the final injury, mice were split into 2 cohorts: sub-acute (SA) and chronic (CH). A total of four groups was formed—SA Sham, SA TBI, CH Sham, CH TBI—with n = 4 mice in each group. SA mice were euthanized at 3 weeks after the last injury while CH were euthanized at 24 weeks post last injury, and their half-cortices were extracted for proteomic analysis.

### Tissue extraction and homogenization

Cortical tissue was homogenized using 500 µL Mammalian Protein Extraction Reagent (MPER) per sample containing 1% EDTA and 1% protease/phosphatase inhibitors cocktail. Samples were sonicated 3 times for 10 min and centrifuged at 10,000 rpm at 4 °C for 10 min. Supernatants were separated and used for further analysis.

### Sample preparation

Frozen tissue homogenates were thawed, and their protein content was determined using a bicinchoninic acid (BCA) assay. Samples were immediately aliquoted to avoid variation in the protein concentrations due to freeze/thaw cycles. A control sample was created by pooling an aliquot containing 60 µg of protein from each of the 48 samples into one Eppendorf tube. Next, six 150 µg aliquots of this control sample were transferred to separate 2 mL Eppendorf tubes. A 10 µL aliquot of a protein standard mixture was added to each control replicate and 100 mM triethylammonium bicarbonate buffer (TEAB) was added appropriately to adjust the final volume to 100 µL. Samples were randomized and divided into six batches with one overlapping repeat sample per batch for between-batch quality control validation. At this stage all batches were frozen at − 20 °C.

Further sample processing was performed in batches and was guided by Thermo Scientific’s instructions for TMT 10-plex Mass Tag Labeling Kits and Reagents, although not strictly followed. Each batch consisted of one control replicate, eight true samples and one repeat sample. Processing of one or two batches of samples was started every 2 days.

Protein reduction and alkylation were accomplished by the addition of 5 µL of 200 mM tris(2-carboxyethyl) phosphine (TCEP) per sample, incubated for 1 h at 55 °C, followed by 5 µL of 375 mM iodoacetamide, incubated for 30 min at room temperature in the dark. The protein was then precipitated with 600 µL of cold acetone, and the process proceeded overnight at − 20 °C. The next day, samples were pelleted by centrifugation at 8000×*g* for 15 min at 4 °C. The supernatant was aspirated, and pellets were allowed to dry for approximately 20 min. The protein was then resuspended in 100 µL of 50 mM TEAB via a 1-h incubation in a thermocycler at 37 °C followed by a 1.5-h bath sonication with intermittent vortexing.

### Protein digestion

Protein was digested first with Lys-C, incubated in a thermocycler at 37 °C for 1 h, and then with Trypsin, incubated in the thermocycler at 37 °C overnight. Samples originally containing 150 µg of protein received 3 µL of (0.25 µg/µL) Lys-C, and those originally containing only 50 µg of protein received 1 µL. Trypsin (0.5 µg/µL) was added in the same manner. Samples were then stored at − 20 °C until ready for peptide labeling.

### Peptide labeling

TMT10plex labeling reagents were brought to room temperature and dissolved in anhydrous isopropanol. For each batch, the combined sample was labeled with TMT-126 and the repeat sample was labeled with TMT-131. Labeling of true samples were randomized. Each sample was combined with one mass-tagging reagent and incubated at room temperature for 1 h. Subsequently, 8 µL of 5% hydroxylamine was added to each sample and incubated for 15 min to quench the reaction. The total volume for each labeled sample was combined into one new Eppendorf tube and stored at − 20 °C. Once all batches reached this stage, the six samples were desalted using Pierce C18 spin columns per product instructions. The instructions include an optional wash step for the removal of excess TMT reagent. The samples were then concentrated on a SpeedVac until sample volumes had been reduced to approximately 25 µL and then stored at − 20 °C.

### Phosphopeptide-enrichment

Phosphopeptide enrichment was performed using TiO_2_ beads using steps modified from Huang et al**.** [35]. Various acidic acetonitrile solutions were used for the sample buffer, and for peptide binding and washing. Ammonia hydrate solutions were used for the elution phase. Sample buffer was added to the samples and the resulting solution was transfer pipetted into an Eppendorf tube containing conditioned TiO_2_ beads. Sample tubes were placed on a shaker for 1 h to facilitate binding of the phosphopeptides. TiO2 beads were collected after a brief spin on a tabletop mini-centrifuge and the supernatant representing the unbound peptide fraction was removed and saved. The beads were washed with a three-step procedure. The supernatants were discarded after these steps. Bound analytes, enriched in phosphopeptides, were eluted in two steps with the addition of ammonia hydrate solutions. The supernatants were saved after each elution step and combined. The phosphopeptide enriched fractions were then concentrated on a SpeedVac until volumes had been reduced to 25–50 µL. Next, an aliquot of 0.1% TFA (aq.) was added to each in preparation for clean-up on Pierce C18 spin columns per product instructions.

### LC/MS/MS analysis

Samples were analyzed on a LC/MS system comprised of an Easy nLC 1000 (Thermo) coupled to a Q Exactive hybrid quadrupole-Orbitrap mass spectrometer with a NanoFlex source (Thermo Scientific). Peptides were trapped on an Acclaim PepMap 100 (75 µm × 20 mm, Thermo Scientific) and desalted. Chromatography was performed on an analytical column (75 µm × 150 mm, Thermo Scientific) packed with C18, 2 µm particles using a 115-min water/acetonitrile reversed-phase gradient method.

The Q Exactive was operated in data-dependent acquisition (DDA) mode. Full scan MS spectra (m/z 400–1800) were acquired in the Orbitrap analyzer with a resolving power of 70,000 (at m/z 200). The fifteen most intense multiply charged ions (z ≥ 2) were sequentially isolated with a 1.0 Da isolation width and fragmented in the collision cell by higher-energy collisional dissociation (HCD). Fragment ions were mass analyzed to a 35,000 resolving power (at m/z 200)*.*

### Peptide and protein identification and quantification

Raw data were processed using Proteome Discoverer software (version 2.1, Thermo Scientific). The MS/MS spectra were searched against a Uniprot mouse protein database (downloaded February 2018) using a target-decoy strategy. Reporter ion intensities were extracted from nonredundant peptide spectral matches (PSMs) and the ratios were determined relative to the combined sample.

### Tissue processing and immunofluorescence

At the time of euthanasia, one hemisphere per mouse was post fixed in a solution of 4% paraformaldehyde at 4 °C for 48 h, dehydrated in graded ethanol solutions, cleared in xylene, and embedded in paraffin. Serial sections (6 µm thick) were cut onto positively charged glass slides and rehydrated in ethanol solutions of decreasing concentrations. Slides were boiled in citrate buffer (pH 6.0) for 7 min for antigen retrieval, transferred to a Sudan Black solution for 15 min to prevent autofluorescence and blocked for 1 h with UltraCruz Blocking Reagent (Santa Cruz: sc-516214). Fluorescent staining was performed with the antibodies for PDE2A (FabGennix: PD2A-101AP) during the overnight incubation. On the next day, secondary antibodies AlexaFluor555 (Thermo Fisher Scientific: A31572) were applied. Slides were mounted with ProLong Gold Antifade 4',6-diamidino-2-phenylindole (DAPI) Mount. Imaging was performed using a confocal microscope (LSM 800 Zeiss) at 20× magnification. Quantification of the fluorescent images was performed using the LSM 800 Zeiss and the number of cells per selected region of interest (ROI) in the cortex was measured.

### Data analysis

The acquired abundance ratios for 5r-mTBI samples were normalized to time-matched controls and used to calculate the up- (> 1.2) or downregulation (< 0.5) of proteins/phosphoproteins. These cutoff levels were chosen based on our previous practice and supported by independent publications [20, 21]. Functional analyses, canonical pathways and networks were generated through the use of IPA (QIAGEN Inc., https://www.qiagenbioinformatics.com/products/ingenuity-pathway-analysis) [36]. The interaction map was constructed using STRING software (version 11.5). The parameters were set to show the full network type indicating both physical and functional associations between the proteins with high confidence interaction score (> 0.7). Edges coloring represents the type of interaction according to the default settings (https://string-db.org/).

## Supplementary Information

**Additional file 1.** Raw data of proteins and phosphoproteins.

**Additional file 2.** Proteins and phosphoproteins in the overlapping pathways.

**Additional file 3.** Molecules driving the overrepresented canonical pathways.

## Data Availability

The datasets used and/or analyzed during the current study are available from the corresponding author on reasonable request.

## Abbreviations

r-mTBI: Repetitive mild traumatic brain injury
AD: Alzheimer’s disease
CTE: Chronic traumatic encephalopathy
MS: Mass Spectrometry
SA: Sub-acute
CH: Chronic
a.r.: Abundance ratio
DDA: Data dependent acquisition
HCD: Higher-energy collisional dissociation
PSM: Peptide specific matches
ROI: Region of interest
IPA: Ingenuity pathway analysis
