# Supplementary figures and images for: Subacute and chronic proteomic and phosphoproteomic analyses of a mouse model of traumatic brain injury at two timepoints and comparison with chronic traumatic encephalopathy in human samples
Source: Mol Brain. 2022 Jul 18;15:62. doi: 10.1186/s13041-022-00945-4 (PMC9290256; doi:10.1186/s13041-022-00945-4)

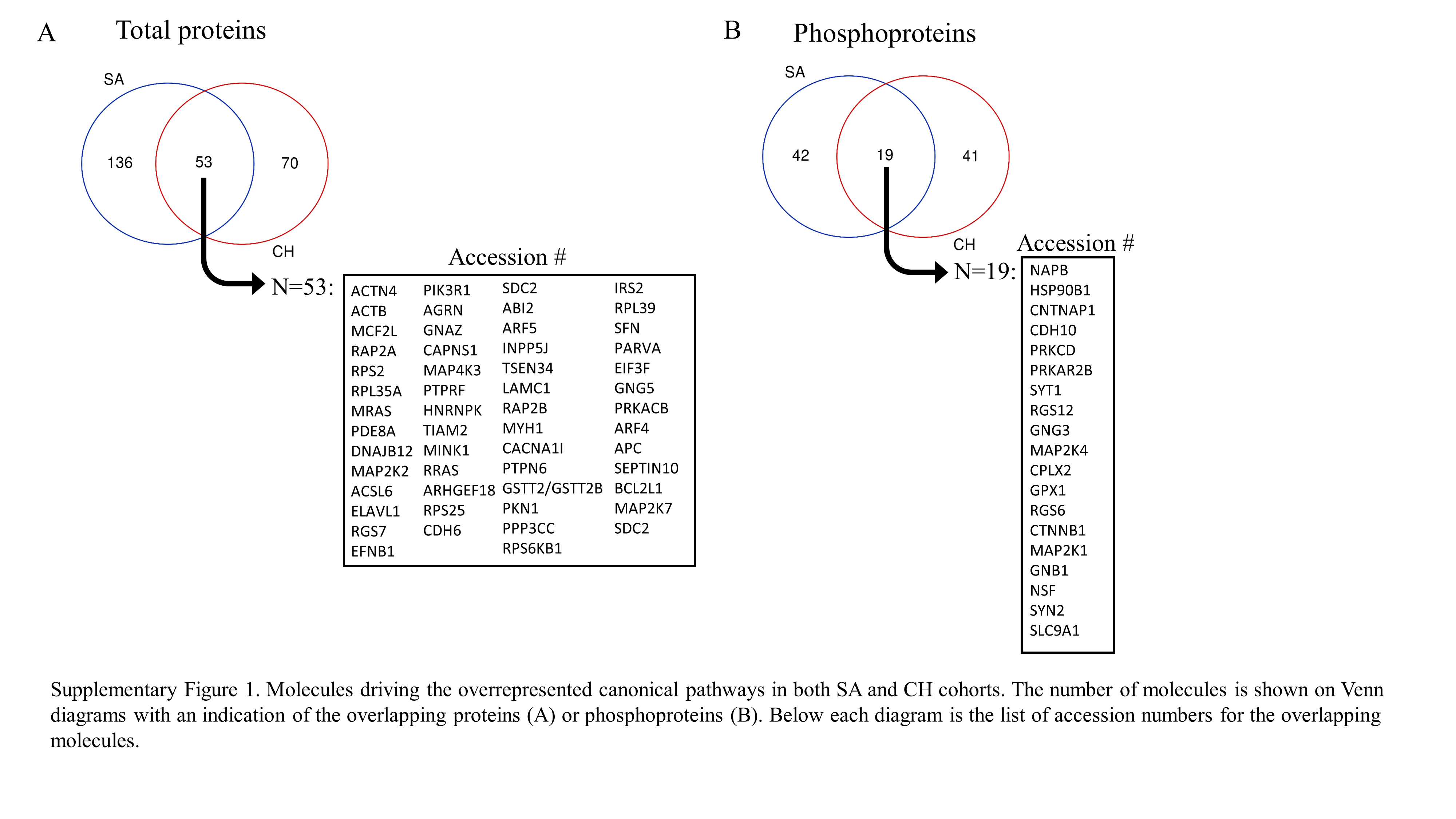

Supplement: Supplementary file 3 — Additional file 3. Molecules driving the overrepresented canonical pathways. [file 13041_2022_945_MOESM3_ESM.png]
